# African migration: trends, patterns, drivers

**DOI:** 10.1186/s40878-015-0015-6

**Published:** 2016-01-22

**Authors:** Marie-Laurence Flahaux, Hein De Haas

**Affiliations:** 1 International Migration Institute, University of Oxford, 3, Mansfield Road, Oxford, OX1 3TB United Kingdom; 2 Faculty of Social & Behavioural Sciences, University of Amsterdam, Nieuwe Achtergracht 166, Amsterdam, 1018 WV The Netherlands

**Keywords:** International migration, Development, Post-colonialism, State formation, Migration determinants, Africa

## Abstract

Africa is often seen as a continent of mass migration and displacement caused by poverty, violent conflict and environmental stress. Yet such perceptions are based on stereotypes rather than theoretically informed empirical research. Drawing on the migration and visa databases from the Determinants of International Migration (DEMIG project) and the Global Bilateral Migration Database (GBMD), this paper explores the evolution and drivers of migration within, towards and from Africa in the post-colonial period. Contradicting common ideas of Africa as a ‘continent on the move’, the analysis shows that intra-African migration intensities have gone down. This may be related to state formation and the related imposition of barriers towards free movement in the wake of decolonisation as well as the concomitant rise of nationalism and inter-state tensions. While African migration remains overwhelmingly intra-continental, since the late 1980s there has been an acceleration and spatial diversification (beyond colonial patterns) of emigration out of Africa to Europe, North America, the Gulf and Asia. This diversification of African emigration seems partly driven by the introduction of visa and other immigration restrictions by European states. Contradicting conventional interpretations of African migration being essentially driven by poverty, violence and underdevelopment, increasing migration out of Africa seems rather to be driven by processes of development and social transformation which have increased Africans’ capabilities and aspirations to migrate, a trend which is likely to continue in the future.

**Electronic supplementary material:**

The online version of this article (doi:10.1186/s40878-015-0015-6) contains supplementary material, which is available to authorized users.

## Introduction

Africa is often seen as a continent of mass displacement and migration caused by poverty and violent conflict. Influenced by media images of massive refugee flows and ‘boat migration’, and alarmist rhetoric of politicians suggesting an impending immigrant invasion, the portrayal of Africa as a ‘continent on the move’ is linked to stereotypical ideas of Africa as a continent of poverty and conflict. In recent years, irregular migration from Africa to Europe has received extensive attention. Sensationalist media reportage and popular discourses give rise to an image of an ‘exodus’ of desperate Africans fleeing poverty at home in search of the European ‘El Dorado’. Millions of Africans are believed to be waiting to cross to Europe at the first opportunity.

The three assumptions underlying such argumentations are that African migration is: high and increasing; mainly directed towards Europe; and driven by poverty and violence. Representations of extreme poverty, starvation, warfare and environmental degradation amalgamate into an image of African misery. Irregular migration occurring from Sub-Saharan Africa and the Maghreb to Europe has also increasingly been defined as a security problem associated with international crime, trafficking and terrorism (Castles, De Haas, & Miller, 2014; Cuttitta, 2007; Goldschmidt, 2006; Lutterbeck, 2006). As the United Nations Office on Drugs and Crime (UNODC) stated, ‘The system of migrant smuggling (…) has become nothing more than a mechanism for robbing and murdering some of the poorest people of the world’ (UNODC, 2006, p. 20).

Not only media and politicians, but also scholars fuel the image of a rising tide of poverty-driven African emigration. For instance, Myers argued that the current flow of ‘environmental refugees’^1^ from Africa to Europe ‘will surely come to be regarded as a trickle when compared with the floods that will ensue in decades ahead’ (Myers, 2005, p.4). This feeds into more general ideas conveyed by scholars such as (Collier, 2013) that we are facing a veritable South–north ‘exodus’ driven by poverty and income gaps, which threatens to spin out of control unless rights of immigrants are curtailed. This reflects a broader tendency in the research literature to cast ‘South-North’ migration as a symptom of development failure (Bakewell, 2008). Based on the common perception that poverty and income gaps between poor and rich countries are the ‘root causes’ of migration, and faced with the ineffectiveness and perverse effects of increased border controls, the frequently proposed long-term ‘solution’ to this phenomenon is to stimulate development in origin countries through aid, trade, or remittances (De Haas, 2007).

The problem is, that such ideas are based on assumption, selective observation or journalistic impressions rather than on sound empirical evidence. The focus of media, policy and research on irregular migration, smuggling, trafficking and the high death toll amongst trans-Mediterranean ‘boat migrants’ reinforce the impression that African migration is essentially directed towards Europe and driven by despair. Since 2000, however, there has been a recent surge in survey- or interview-based studies on contemporary African migrations (Bakewell & Jόnsson, 2011; Berriane & De Haas, 2012; Bilger & Kraler, 2005; Bredeloup & Pliez, 2005; De Bruijn, Van Dijk & Foeken, 2001; Lessault & Beauchemin, 2009; Lessault & Flahaux, 2013; Pian, 2009; Schielke & Graw, 2012; Schoorl et al., 2000; Whitehouse, 2012). These studies have shed light on the diversity of African migration. Several studies have shown that most African migrations are not directed towards Europe, but towards other African countries (Schoumaker et al., 2015; Sander & Maimbo, 2003) and that those moving out of Africa do not only move to Europe but also to the Gulf countries and the Americas (Bakewell & De Haas, 2007).

Contradicting common idea that African emigration is essentially about irregular movement, previous research has suggested that most Africans migrate out of the continent in possession of valid passports, visas and other travel documentation (cf. Schoorl et al. 2000, Heering, Esveldt, Groenewold, van der Erf, Bosch, de Valk, and de Bruijn, 2000). More generally, recent scholarship has started to question the implicit assumption that African migration is ‘exceptional’ and essentially different from migration elsewhere. More and more micro-evidence emerges indicating that most Africans migrate for family, work or study (Schoumaker et al., 2015) (Bakewell & Jόnsson, 2011), as is the case in other world regions. In a recent study on the Great Lakes region, (Bakewell & Bonfiglio, 2013) argued that although it would be impossible to deny the importance of conflict as a cause of (forced) migration in the region, that it would ‘equally wrong to neglect the on-going, perhaps mundane social processes that drive mobility, such as the search for an education, a spouse or a better life in the city’ (Bakewell & Bonfiglio, 2013, p. 4). According to official data, refugees and ‘people in refugee-like situations’ represented 2.4 million or 14 per cent of international migrants in Africa (UNHCR, 2011). Although this is a higher proportion than in other regions, this implies that about 86 per cent of international migration within Africa is *not* primarily related to conflict.

Notwithstanding the increasing availability of survey- and interview-based micro-level data on African migration, data availability remains extremely patchy and is generally focused on migration to Europe from a limited number of better-researched African countries, such as Morocco, Senegal, Ghana and South Africa. What has been particularly lacking so far, is macro-data that allows to map the overall evolution of the migration patterns from, to and within Africa over the past decades. This is not only important to gain a more fundamental insight into the factual evolution of African migration and to verify the validity of common perception of massive and increasing African migration, but it would also allow to contribute to the scholarly debate on the determinants of migration. On the one hand, this pertains to the debate on how development affect human mobility in which scholars have challenged conventional push-pull models by arguing that, particularly in poor societies, development increases rather than decreases levels of migration (Clemens, 2014; De Haas, 2010; Skeldon, 1997). On the other hand, conventional accounts of African migration tend to ignore the role of African states in shaping migration. This reflects the more general Eurocentric (destination-country) focus of migration research.

In order to fill these research gaps and gain a better understanding of the nature and causes of African migration, this paper analyses the evolution of migrations within, towards and from Africa in the post-colonial era, and explores the main factors explaining changes in the volume and the direction of these migrations. It will do so by drawing on new longitudinal databases containing data on migration stocks and flows, which have significantly extended the capacity to perform such analyses. Before embarking upon the empirical analysis, however, we will further explore the theoretical arguments that compel us to critically rethink role of development and states in migration processes.

## Theoretical background: the drivers of migration

### Conceptualising the role of development

The idea that much African migration is essentially driven by poverty ignores evidence that demographic and economic transitions and ‘development’ in poor countries are generally associated to *increasing* rather than decreasing levels of mobility and migration and that the relation between development and migration is fundamentally non-linear. This argument was originally put forward by (Zelinsky, 1971) in his *Hypothesis of the Mobility Transition.* Zelinsky argued that processes of modernisation and economic development have historically coincided with increasing rural-to-urban migration followed by a subsequent *increase* in emigration. When societies become wealthy emigration decreases and immigration increases, leading to a mobility or migration transition, in which countries gradually transform from countries of net emigration into countries of net immigration. These ideas about a ‘migration transition theory’ have been further developed (De Haas, 2010; Martin & Taylor, 1996; Skeldon, 1997) and empirically tested using historical (Hatton & Williamson, 1998) and contemporary (Clemens, 2014; Czaika & De Haas, 2012; De Haas, 2010) data sources.

Such insights turn the predictions of conventional ‘push-pull’ models or neoclassical theories that predict that migration decreases as societies develop and income and other geographical opportunity gaps decrease upside down. In reality most migrants do not move from the poorest to the wealthiest countries, and the poorest countries tend to have lower levels of emigration than middle-income and wealthier countries. To understand why development is generally associated to more migration, it is important to move beyond views of (African) migrants as objects which are passively pushed around by external ‘push’ factors such as poverty, demographic pressure, violent conflict or environmental degradation, analogous to the way physical objects are attracted or repelled by gravitational or electromagnetic forces.

Such ‘push-pull’ views, however, ignore that people will only migrate if they have the ambitions and resources to make this happen. We can see migration as a function of people’s *aspirations* and *capabilities* to migrate (De Haas, 2011; 2014). This idea helps us to understand why development is often associated to increased levels of migratory as well as non-migratory mobility (such as commuting, tourism and business travel). Development processes typically expand people’s access to material resources, social networks, education, media and knowledge. At the same time, improvements in infrastructure and transportation, which usually accompany development, make travel less costly and risky, enabling migration over increasing distances.

Yet increased migration capabilities do not automatically lead to migration if people do not aspire to do so. Migration aspirations depend on people’s more general life aspirations and their perceptions of the extent to which these aspirations can be fulfilled ‘here’ and ‘there’. Both these aspirations and perceptions about geographical opportunities are highly subjective and likely to change under the influence of social and cultural change. Improved access to information and exposure to other (wealthy and/or ‘Western’) lifestyles conveyed through education, media and advertising tend to change people’s perceptions of the ‘good life’ alongside increasing material aspirations and a growing appetite for consumer goods. The crux is that when ‘development’ occurs in poor and marginal countries and areas, aspirations and capabilities to migrate tend to increase simultaneously, explaining the paradoxical phenomenon of development driven emigration booms.

Although poor people do also migrate, they tend to do so less often, and if they migrate, they tend to do so overall smaller distances. This also seems to explain why the skilled and relatively wealth are overrepresented among long-distance international migrants. This particularly holds when border controls and immigration restrictions increase the costs and risks of migrating to wealthy countries. We can therefore also expect emigration to become less selective if societies as a whole become wealthier and more developed, as this will also lift relatively poor people above the material threshold needed to migrate internationally, initially to neighbouring countries but increasingly also overseas.

If societies get wealthier, more people can imagine a future within their own country and emigration is likely to decrease. Wealthy societies, however, remain highly mobile and migratory. This is partly related to the high levels of educational and occupational specialisation, and overall organisational complexity of modern societies, which requires people to move within and across borders to fulfil the desire to match qualifications and personal preferences with labour market and social opportunities. The higher skilled therefore tend to migrate more and over larger distances. This shows that it is illusionary to think that large-scale migration is somehow a temporary phenomenon that will disappear once – an equally illusionary – equilibrium is achieved, as conventional push-pull models would predict. We therefore need to refute popular ‘push-pull’ models, as they lead to misleading analyses on the nature, causes and future of migration.

### Conceptualising the role of states in migration processes

Common accounts of African migration are also characterised by either an ignorance or a weak theorisation of the role of African states in migration processes, which reflects a broader ‘receiving country bias’ in migration research, which obscures the role of origin states (Vezzoli, Villares-Varela, & De Haas, 2014). This also ignores the fact that poor countries are *also* destination countries. While the increasing immigration restrictions and border controls put in place by European destination states have received ample attention, the role of colonial and post-colonial African states in shaping migration processes is poorly understood. This is a major research gap. First, colonial occupation and concomitant practices of the slave trade and the systematic use of forced labour and recruitment have in many ways shaped contemporary migration patterns within and from the continent (Cohen, 1987). During the period of colonial liberation, millions of people fled conflicts with colonial powers reluctant to relinquish control (Algeria, Kenya, etc.) or with white settler groups determined to cling to their privileges (eg Zimbabwe, South Africa) (Castles, De Haas, & Miller, 2014). Yet, the defeat of old-style colonialism and the establishment of independent states often did not necessarily mean a return to peaceful conditions (Castles et al. 2014, De Haas, & Miller, 2014). During the Cold War, East and West fought proxy wars in Africa while backing undemocratic regimes and supporting the toppling of democratic governments. Political and economic pressures, arms supplies, mercenaries and even direct military intervention were factors contributing to new conflicts or the continuation of old ones (Zolberg, Suhrke, and Aguayo, 1989). For instance, struggles for domination in Angola, Mozambique and Ethiopia involved massive external involvement, with great human costs for local populations (Bakewell, 2000; Castles, De Haas, & Miller, 2014).

Decolonisation also heralded a phase of state formation, in which newly established African states have endeavoured to instil a sense of national unity in ethnically diverse societies, which often created considerable internal tensions and has regularly erupted in violent conflicts (cf. Davidson, 1992). State formation processes and concomitant conflicts have theoretically uncertain effects on population mobility, which are as yet poorly understood. On the one hand, instability, uncertainty and conflict may provide incentives for people to leave. On the other hand, it may also provide incentives for people to stay in order to provide protection for their families. In the same vein, people living under authoritarian regime may more often wish to migrate, but authoritarian states may also have a higher willingness and capacity to control and restrict emigration. This may explain why a recent analysis of global migrant stock data found a robustly *positive* relationship between the level of political freedom and emigration (De Haas, 2010).

Although the formation of nation states can go along with increasing migration (cf. Skeldon, 1997) either through conflict, infrastructure, or policies that encourage emigration as a means to decrease unemployment, generate remittances, and decrease dissatisfaction, increased nationalism, anti-colonial sentiment, xenophobia and protectionism associated to the same state formation processes has also compelled several African governments (such as Algeria, Egypt, and Côte d’Ivoire) to discourage the emigration of their own populations to control emigration or out of the fear of a ‘brain drain’ and to restrict the immigration of foreigners (Natter, 2014; Samers, 1997; Zohry & Harrell-Bond, 2003). Particularly socialist and/or nationalist governments have traditionally been anti-emigration. Processes of state formation may also have increased the urge among leaders of newly established states to assert national sovereignty by introducing immigration restrictions and border controls and to portray immigrants as a threat to sovereignty, security and ethnic homogeneity or stability in a bid to rally political support. In this context, African governments have frequently resorted to deportations. For instance, (Adepoju, 2001) counted 23 mass expulsions of migrants conducted by 16 different African states between 1958 and 1996.

Political tensions and military conflict pushed many countries to attempt to seal off their mutual borders, such as between the Frontline States in Southern Africa with South Africa as part of the anti-Apartheid struggle and between Morocco and Algeria as part of the conflict around the Western Sahara. Particularly, socialist states such as Algeria and Egypt (under Nasser) saw large-scale emigration as a source of brain drain and a threat to sovereignty, and therefore tried to curb emigration (Collyer, 2003; Fargues, 2004, p. 1360; Natter, 2014; Sell, 1988). This shows that states can both facilitate and constrain migration in various direct and indirect – and therefore complex – ways, and that this relation needs in-depth empirical inquiry to be better understood.

This paper uses new data to explore the *volume* and the *direction* of migration from, within and to Africa in order to contribute to broader debates on the role of development and states in migration processes. Table 1 summarises the theory-derived ideas about how broader processes of development and (post-colonial) state formation affect migration. Although the migrant population (‘stock’) data this paper draws from enables us to map the evolution of migration patterns, more comprehensive flow data is necessary to formally test hypotheses on migration determinants. Rather than providing formal tests for these ideas, this paper assesses the extent to which these ideas seem to hold in the light of African migration realities and to formulate hypotheses which can be further evaluated by future research.

**Table 1:** Dimensions of analysis and theory-derived ideas on migration determinants

	Role of development	Role of states
Volume	Emigration initially increases with development, to decrease at higher development levels.	Autocratic and nationalist governments are better able and willing to reduce levels of emigration and immigration.
Direction	Development leads to an increasing proportion of populations to migrate to other continents.	Post-colonial relations stimulate migration to former colonisers, although this ‘colonial echo’ decreases over time.

## Data

African migration research is haunted by the lack of reliable official data and the absence of appropriate sampling frameworks in the form of census or survey data. Although these problems are far from resolved, recently, the availability of new migration databases has significantly expanded the scope to conduct analyses on migration from, to and within Africa^2^.

The Global Bilateral Migration Database (GBMD), which was released by the World Bank, contains bilateral migration population (‘stock’) data for 226 countries, major territories and dependencies for each decade from 1960 to 2000 (Özden, Parsons, Schiff & Walmsley, 2011). This database is based on census data and population register records (when census data were not available). While the release of this database has drastically increased the potential to assess long-term migration trends, it has some limitations. For instance, immigration is likely to be underestimated for countries defining migration on the basis of ‘citizenship’ rather than ‘birth’ because of naturalisation. Moreover, irregular migration is generally not taken into account and if data was missing some values have been estimated. Because the 1970 data turned out to be inconsistent, we decided only use 20 year intervals (ie, 1960, 1980 and 2000) to study the evolution of African migration.

The recently completed DEMIG C2C (‘country-to-country’) migration flow database (which has been part of the Determinants of International Migration project) has drastically expanded our ability to perform detailed analysis of recent patterns and trends of African migration to and from Europe, North America and Oceania. DEMIG C2C database covers bilateral migration flows for 34 reporting countries from and to a broad range of origin countries over the 1946–2011 period (see Vezzoli, Villares-Varela, & De Haas, 2014). This database also has its limitations. In general, migration flow data is generally less reliable than stock data and its coverage is patchier. For instance, flow data are not available for some important destination countries such as the United Kingdom. When flow data are based on population registers they are not always comparable because the registration criteria (such as duration of stay) can vary considerably across countries.

This paper will use these new data sources to analyses the evolution of migration patterns within, towards and from Africa in the 1960–2010 period. The analysis of the GBMD stock data will give a global and long-term perspective of the evolution of these migrations. The analysis of the DEMIG C2C flow data will give better insights into the recent evolution of African migration to Europe, North America and Oceania, as well as patterns of diversification in terms of destination and origin countries. To assess the level of development of African countries, world development indicators available on the World Bank website will be used (GDP, mortality rate of children under five years old, fertility rate and proportion of rural population). To estimate the restrictiveness of immigration policies of African and non-African states, the paper will draw on the DEMIG VISA database, which is a global panel of bilateral travel visa requirements covering the 1973–2013 period (De Haas & Villares-Varela, 2014 forthcoming).

## Volume and direction of African migration

### The evolution of the migration intensity from, to and within Africa since 1960

The volume of migration from, to and within Africa may be measured in absolute or relative numbers. As Table 2 shows, total stocks of migration from Africa to the rest of the world and within Africa have increased between 1960, 1980 and 2000, while migration from the rest of the world to Africa has decreased in absolute numbers.

**Table 2:** Estimated total stocks of migration from, to, and within Africa

	From Africa to the rest of the world	From the rest of the world to Africa	Within Africa
1960	1 830 776	2 811 930	6 176 385
1980	5 418 096	1 872 502	7 966 359
2000	8 734 478	1 532 746	10 500 000

Yet, to measure the intensity of emigration it is more appropriate to calculate the volume of migration in relation to the size of the population born in origin countries or destination countries. To measure *emigration intensity* we divided the numbers of emigrants from each country by the population born in the same country. In order to measure the relative importance of immigration from particular African or non-African countries in destination countries, we calculated *immigration intensity* by dividing the number of migrants from particular origin countries (African and non-African) in each destination country by the population born in each of these countries. It is important to emphasise that these measures for emigration and immigration intensities are based on migrant stock data, and should therefore not be confused with migration rates which are usually based on annual flow data. Migration intensities give a general estimate of the intensity of migration to and from particular countries in the recent past.

#### African emigration intensity

**Fig. 1:**
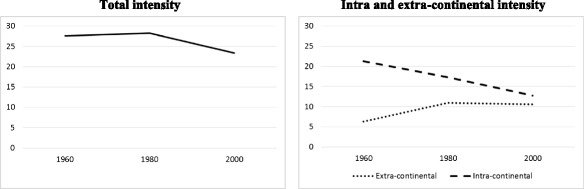
Evolution of emigration intensity from African countries (per 1000; weighted by population size). Source: Global Bilateral Migration Database

Our analysis confirms that the majority of African migrants move within the continent. Figure 1 shows trends in the average emigration intensity. All results have been weighed for population size of each country. This reveals two striking trends. While intra-African emigration has shown a clearly *declining* trend between 1960 and 2000, extra-continental emigration, albeit much lower, shows an *increasing* trend, particularly between 1960 and 1980. In 2000, African countries had on average 2.3 % of their citizens officially living abroad, down from 2.8 % in 1960. While average extra-continental emigrant intensity went up from 0.6 % in 1960 to 1.1 % in 2000, intra-continental emigrant intensities went down from 2.1 to 1.3 %. The robustly declining trend of intra-continental migration may seem puzzling at first sight, as it contradicts popular accounts of Africa as a continent ‘on the move’.

**Fig. 2:**
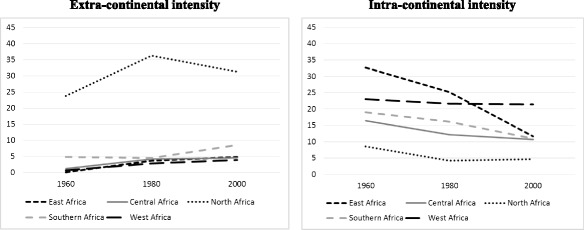
Evolution of emigration intensity by African region (per 1000; weighted by population size). Source: Global Bilateral Migration Database

Figure 2 shows that the increase in extra-continental migration between 1960 and 1980 from Africa mainly reflects the high emigration intensity from Maghreb countries, but not from other regions^3^, where the emigration intensity to non-African destinations stays below one percent. The decrease in intra-continental mobility has been particularly stark in East Africa and to a lesser extent in Southern and Central and North Africa, while emigration from West Africa within the continent has remained on a consistently high level (see Fig. 2).

**Fig. 3:**
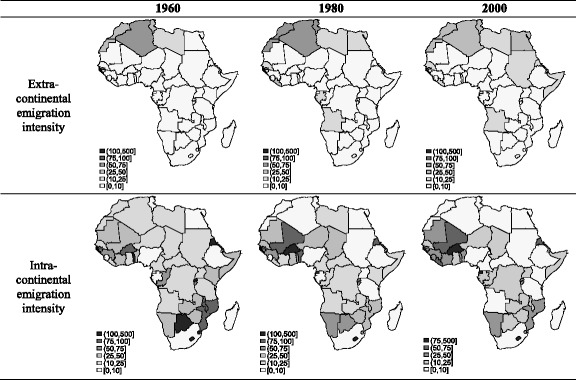
Evolution of emigration intensity from African countries (emigrants per 1000 people born in each country). Source: Global Bilateral Migration Database

Figure 3 depicts the emigration intensity from the different African countries. The maps corroborate that African emigration is first and foremost about migration within the continent. Intra-continental emigration intensities tend to be highest in inland West-African countries (such as Mali and Burkina Faso), some Southern African countries and small states such as Lesotho and Eritrea, and tend to be low in the Maghreb (where most people migrate to Europe) and populous countries such as Nigeria, Egypt, and South Africa.

The paradox of declining intra-African migration might partly be explained by the fact that decolonisation and the concomitant antagonism between newly created states may have indeed increased intra-continental barriers to movement. The comparatively high migration levels in West-Africa seem linked to the fact that this region contains many smaller countries both in population and in land surface. In countries with small populations, more migration is likely to spill over national borders, explaining why small countries have on average higher emigration intensities (De Haas, 2010). Other factors may include that many ethnic groups are spread in several West-African countries which provide strong network connections across borders, as well as the fact that under colonial rule strong (generally coast-bound) migration patterns were already established. A final factor may be that there is visa-free movement between ECOWAS (Economic Community of West African States) countries (cf. OECD, 2006).

Figure 3 also shows that extra-continental emigration intensities are highest in North African countries. Our analysis, however, suggests that emigration from the Maghreb region are declining to a certain extent, which *may* indicate that they are over the peak of their ‘migration transition’, which confirms more detailed studies of Maghreb emigration (Natter, 2014).

**Fig. 4:**
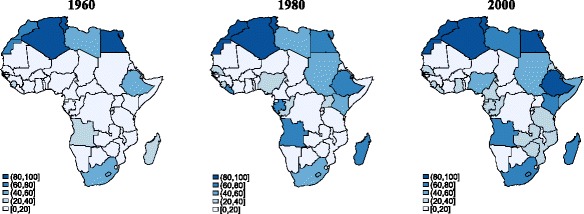
Extra-continental emigration intensity as a proportion of total emigration intensity (percentage). Source: Global Bilateral Migration Database

Figure 4 depicts the level of extra-continental migration (out of Africa) as a proportion of total emigration. It shows some clear patterns. First, in 1960 North Africa was the only region where extra-continental migration was higher than intra-continental migration, with the exception of Ethiopia and South Africa^4^. The Maghreb is Africa’s emigration region *par excellence*. This is related to its geographical proximity to Europe, their strong colonial and post-colonial links to France and the labour recruitment agreements these countries signed with European countries since the 1960s (De Haas, 2008; Natter, 2014). Egypt has weaker ties to Europe, but since Sadat’s *infitah* (open door) policies of the 1970s massive emigration has occurred oil-producing Libya and the Gulf States (Sell, 1988; Zohry & Harrell-Bond, 2003).

Second, some other countries, such as Angola and Ethiopia (and to some extent Somalia, Sudan, Kenya and Uganda) stand out as countries with strong extra-continental connections but weak regional migratory connections. This is possibly related to conflict and long-distance networks created as a consequence of refugee resettlement and (in the case of Angola) colonial ties. Third, South Africa has relatively low emigration intensity, but as far as people emigrate it is overwhelmingly out-of-the-continent. Fourth, the urban coastal zones of a number of relatively prosperous West-African countries (Ghana, Nigeria, Senegal) form an emergent zone of increasing extra-continental emigration (with the notable exception of Côte d’Ivoire). More generally, it seems that countries with a high proportion of extra-continental emigration intensity are those with comparatively higher levels of economic development.

#### Immigration intensity within Africa

**Fig. 5:**
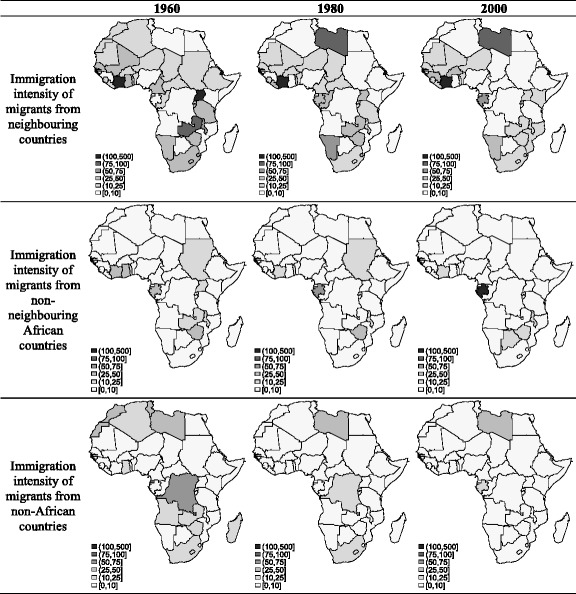
Evolution of immigration intensity from African countries (immigrants per 1000 inhabitants). Source: Global Bilateral Migration Database

Figure 5 shows the evolution of immigration intensity in African countries for ‘neighbouring African countries’, ‘non-neighbouring African countries’ and ‘non-African countries’. The results mirror the findings on emigration intensity confirming that the bulk of African migration is contained within the continent and, more specifically, occurs between neighbouring countries.

The maps indicate that there has been an increasing concentration of African migrant populations in particular countries or clusters of countries. The overall pattern has been coast-bound migration from more marginal inland countries and areas. Post-independence migration has been increasingly about urbanisation and the concomitant transfer of population from inland, marginal rural areas to fertile agricultural areas, towns and cities which are often located in coastal areas. These patterns do not only exist between countries (such as between Burkina Faso and Côte d’Ivoire) but also within countries (such as north–south migration within West African countries or south–north migration in Egypt). While Coastal West Africa and Southern Africa are larger, historically well-established immigration zones, the migration hubs in Djibouti and the oil economies of Gabon (from West and Central Africa) and Libya (from neighbouring countries) have more recently risen as major African migration destinations. The number of countries, however, with high immigration intensity has decreased over time, which reflects a general trend of decreasing intra-African migration.

While declining intra-African migration is not a uniform process, it fundamentally questions popular accounts of African migration as high and rapidly increasing. More research into the causes of declining intra-African migration intensity is required, hopefully with improved data. One possible explanation is that independence, the attempts to create nation-states (cf. Davidson, 1992) and the concomitant drive to assert restrictive border regimes may have curbed migration to a certain extent. Between 1950 and 1975, the large majority of African countries gained independence. Under colonial rule, mobility between countries, particularly when they were under the same colonial authority, may have been easier than after decolonisation, when states may have been keen to assert their newly acquired sovereignty by demarcating borders. Particularly when states embarked upon a more protectionist political and economic track, this may have coincided with increasing immigration restrictions and nationalism.

For instance, in the 1950s and 1960s many West African migrants moved to Ghana. After the 1966 coup in Ghana and the concomitant economic decline, the immigrant community became a scapegoat. In 1969 the Ghanaian government enacted the Aliens Compliance Order, which led to a mass expulsion of about 200,000 migrants, mainly from Nigeria, Togo, Burkina Faso and Niger (Van Hear, 1998, p. 73–74). With Nigeria’s new oil wealth after 1973, millions of Ghanaians and other West Africans sought work there. But corruption and misguided economic policies precipitated a crisis, and in 1983–1985 an estimated two million low-skilled West Africans were deported from Nigeria, including over one million Ghanaians (Van Hear, 1998, p. 73–74).

Although African states are often said to have very porous borders, there has definitely been a drive to make them less porous as part of a larger quest to assert the sovereignty of African over their territory and the populations inhabiting that territory. Although regional organisations such as the Economic Community of West African States (ECOWAS), the South African Development Community (SADC), the East African Community (EAC) and the Common Market for East and Southern Africa (COMESA) have introduced rules for free movement of nationals between their member states, these agreements have generally been poorly implemented or contradicted by the restrictive policies and practices of member states (Adepoju, 2001; Ricca, 1990). Migrant rights are not always protected, and as elsewhere in the world, migrants are often scapegoated and mass deportations have regularly occurred, particularly in times of economic crisis (Castles, De Haas, & Miller, 2014).

Figure 5 also highlights that there has also been a stark decrease in immigrant population originating from non-African countries in Africa. This sharp decrease in the presence of non-African immigrants in Africa, particularly in North and Central Africa seems to partly reflect the departure of colonial administrators and settlers after independence, such as the massive departure of French colons from Algeria. But it also reflects a sharp decrease in immigration from Europe, the Indian subcontinent (to East and Southern Africa) and Lebanon (mainly to West Africa). In some cases it reflects policies of ethnic cleansing, such as the deportation of Ugandan residents of Indian by the Idi Amin regime. In 2000, oil-rich Libya and Gabon remain among the rare countries that attract significant shares of non-African immigrants.

### The geographical diversification of African emigration

#### General migration trends

**Fig. 6:**
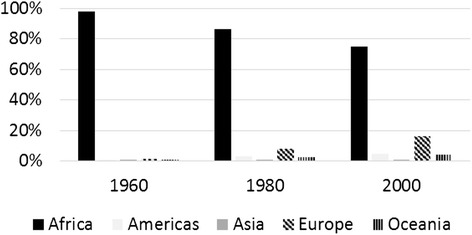
Destinations of African migrants in 1960, 1980 and 2000. Source: Global Bilateral Migration Database

Figure 6 shows that although African migrants are still overwhelmingly located in African countries, the proportion of Africans living in (North) America and, particularly, Europe has increased. As we will see, because these are *stock* data, this underestimates the actual rate of increase of migration *flows*. The figures in Additional file 2 break these data down on the regional level and show that this increase in extra-continental has been general, although with strong regional variations. After Africa, Europe is the second continent of destination for migrants from West, East, Southern and Central Africa. Migrants from Southern Africa, however, also migrate to America and Oceania in significant numbers. Finally, the main destinations of North African migrants are Europe and Asia (Gulf countries), followed by Africa. This confirms that, with the exception of North Africa, migration out of Africa has been historically low.

**Fig. 7:**
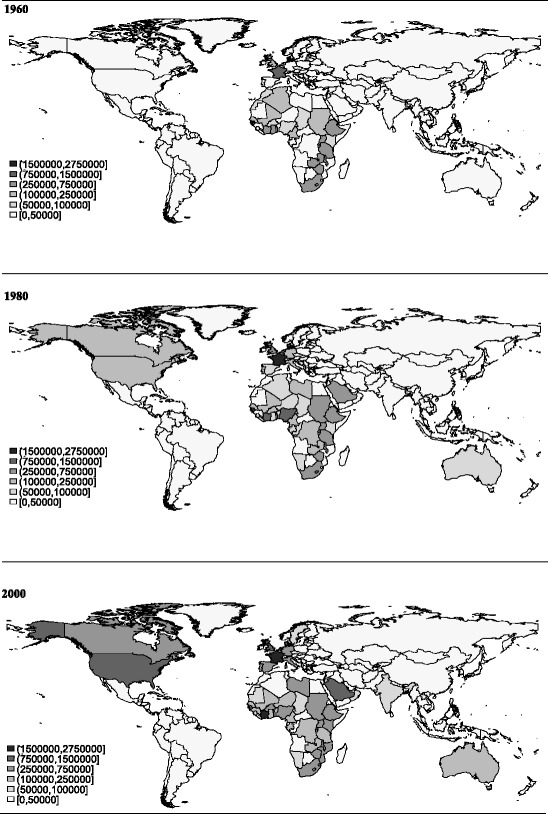
Absolute numbers of African migrants in destination countries in 1960, 1980 and 2000. Source: Global Bilateral Migration Database

In order to obtain an overview of the spread of African emigration in terms of destination countries, Fig. 7 depicts the total numbers of African migrants in the world in 1960, 1980 and 2000. Besides corroborating that the bulk of African migrants move within the continent, it also shows that the number of African living in Europe, North America, and other countries such as Australia and India has been increasing. In 1960 most extra-continental migrants lived in France and the United Kingdom, which is not surprising given their position as the dominant colonisers of the African continent. Since then there has been a clear patterns of diversifications of European destinations, with countries such as Germany, the Netherlands, Italy, Portugal and Spain becoming important destinations. We also see a clear increase and diversification of African emigration to non-European destinations, and particularly towards Saudi Arabia, Australia, USA and Canada. This overall pattern of diversification seems to indicate of a declining ‘colonial echoe’ in African emigration.

#### Recent African migration to Europe, North America and Oceania

While the GBMD is useful to identify long-term migration trends ad patterns, there are less useful for analysing more recent trends in African migration. First, stock data that are very much a ‘legacy of the past’ that approximate net migration over several decades, which means that recent changes in migration patterns only show up after a long period. Second, the last census round included in the GBMD dates back to 2000. In order to substantiate and complement the preceding analysis, we analyse recent flow data from the DEMIG C2C database, which is particularly useful to uncover recent trends of extra-continental African migration.

**Fig. 8:**
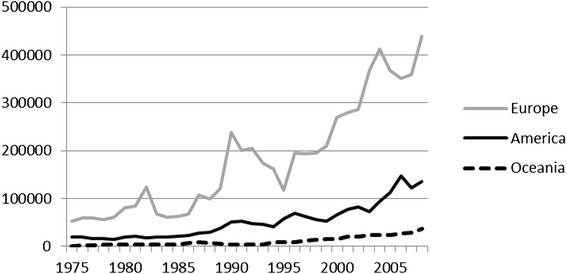
African migration flows to selected OECD countries by continent of destination^5^. Source: DEMIG C2C database

**Fig. 9:**
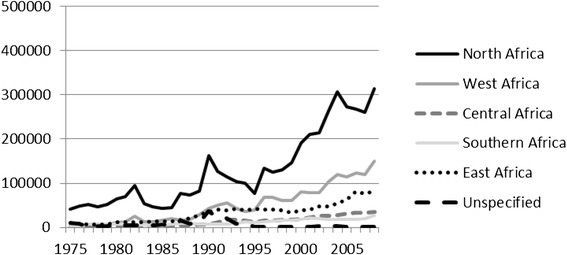
Evolution of extra-continental African emigration by region of origin^6^. Source: DEMIG C2C database

**Fig. 10:**
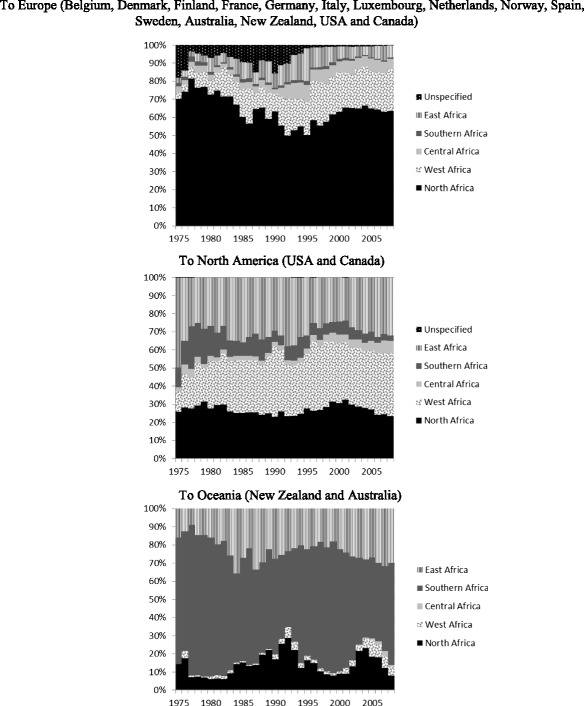
Evolution of the regions of origin of African migrants by continent of destination. Source: DEMIG C2C database

Figure 8 shows that there has been a recent acceleration and diversification of extra-continental migration from sub-Saharan Africa, particularly to the United States and Canada. Figure 9 gives further detail on the evolution of non-African migration destination by region of origin. While North Africa is still overrepresented in extra-continental emigration, the share of other regions, particularly West and East Africa is increasing. Figure 10 reveals that North- and West- Africans are predominant in African migration to Europe, which seems related to the relative proximity to Europe and the historical role of labour recruitment in Francophone countries in the Maghreb but also in Senegal and Mali.

African migration to the Americas (US and Canada) is much more diverse, although Southern and Central Africa are relatively underrepresented in this migration. This may be partly explained by the absence of colonial ties, the effect of the US visa lottery and the comparative openness of the US and particularly Canada, for high-skilled high immigration, which may have enabled more diffuse immigration patterns in terms of origin countries. The United States is becoming an increasingly important destination among extra-continental migration, in particular from West Africa. African migration to Oceania (mainly Australia and to some extent New Zealand) is small but increasing, and dominated by Southern Africans, which seems to largely follow British colonial.

The graphs presented in Additional file 3 show that Europe in the main destination outside Africa for migration flows from North, Central and West Africa, while the extra-continental migration flows from East Africa are increasingly directed toward America, and those from Southern Africa toward Oceania and America.

## Exploring developmental and policy drivers of African migration

### The role of development processes

**Fig. 11:**
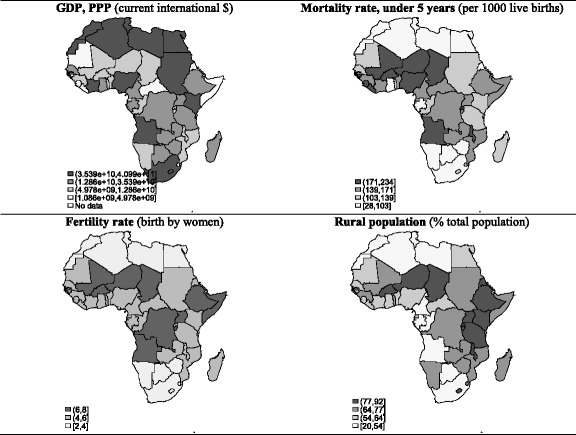
Economic and demographic situation of African countries in 2000. Source: World Development Indicators, World Bank

There seems to be a rather clear relation between levels of socio-economic development and the volume and geographical orientation of African emigration. More marginal, poorer or landlocked countries tend to have lower absolute and relative levels of extra-continental migration, and their migration is primarily directed towards other African countries. This seems to confirm migration transition theory, according to which materially poor populations of the least developed countries have less capabilities to move, and when they move, they tend to move over shorter distances, either internally, or to other African countries. Figure 4 shows that the countries with relatively high extra-continental migration are also the countries that are located on the coast, that are more urbanised, have a higher GDP per capita, and are more advanced in the demographic transition as indicated by lower mortality and lower fertility (see Fig. 11). This seems to confirm the hypothesis of transition theories pioneered by (Zelinsky, 1971).

There are some notable exception to these overall patterns. While Ethiopia has a very low estimated emigrant intensity (0.4 % in 2000), emigration used to be mainly directed at North America and Europe, at least until the onset of larger-scale migration to the Gulf in the 2000s. This may be related to the predominance of refugee flows from Ethiopia and the fact that many refugees have been resettled in North America and Europe. Another, albeit very different, exception to this general pattern is Côte d’Ivoire, which is a major intra-African destination, but sees few of its own citizens leaving, either within or outside Africa. This is very different from patterns in South Africa, which attracts migrants from elsewhere in Africa but is also an important source of emigration out of the continent, highlighting its major role as a hub linking intra- and extra-African migration.

This seems linked to the fact that South Africa (and, to a certain extent, other countries in Southern Africa and Kenya and Uganda) has witnessed large-scale settlement from the UK, Netherlands and other European countries and has also witnessed the large-scale arrival of populations from the Indian subcontinent and China over the colonial period. As a consequence of this history of settlement, descendants of European and Asian immigrants have retained strong transnational ties with origin and other settlement countries, such as the UK, US, Australia, India, Pakistan and China. The concomitant intensity of transnational social, economic and political ties now facilitate migration out of the continent. Côte d’Ivoire, by contrast, lacks a history of large-scale European settlement and is therefore less connected to inter-continental migration systems.

### The role of states and policies

While levels of development seem to clearly affect immigration and emigration volumes and the distance of migration, state policies also play an important role. We argue that, in order to explain the comparatively low and declining intra-African migration intensity and low immigration towards Africa, xenophobia and immigration restrictions imposed by African states may play a role. In addition, we hypothesise that the diversification of African migration beyond Europe is partly be driven by increasingly immigration restrictions by former colonising countries (mainly France and the UK) and other European destinations. This may have prompted increasing numbers of Africans, particularly those possessing the education and skills allowing them to obtain visas, to explore new destinations in North America, Oceania and elsewhere. There is indeed some evidence of increasing migration of Africans in search of work, education and business opportunities to fast growing economies such as China (Ghosh, 2010), India, Russia, Turkey, Japan, Brazil and Argentina (Henao, 2009).

The role of policies in shaping migration flows in general and in Africa in particular has been understudied, partly due to the lack of appropriate policy data. This section uses new panel data from the DEMIG VISA database (De Haas and Villares-Varela, 2014 forthcoming) to construct measures of relative immigration restrictiveness of African and non-African destinations (broken down by major regional blocks) between 1973 and 2014. This *visa restrictiveness index* has been calculated by computing the percentage of origin countries that need a travel visa to enter destination country for every year. For the graphs, we subsequently calculated the average value for all destination or origin countries within the regional aggregated of interest for every year.

**Fig. 12:**
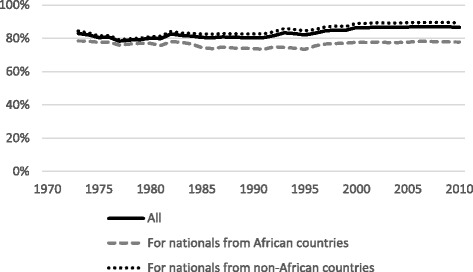
Visa restrictiveness of African countries (1973–2013). Source: DEMIG VISA database

Figure 12 shows the *visa restrictiveness indices* of African countries specified for African and non-African nationals. The figure shows that the level of border restrictions of African countries is rather high and has in fact shown an increasing trend, particularly since the late 1980s. In 2013, on average, about 90 % of nationals from non-African countries needed a visa to enter African countries, while on average 78 % of Africans needed a visa to enter another African country^7^. This is substantially higher than the global average of bilateral visa requirements of 65 % reported in (Czaika & De Haas, 2014), which seems to confirm that African states are rather closed towards the free movement of people.

**Fig. 13:**
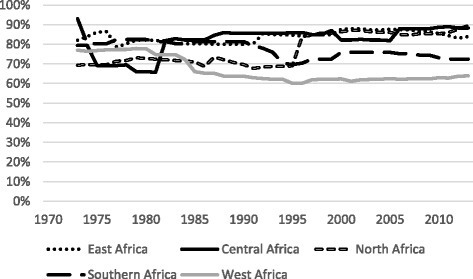
Visa restrictiveness of African countries for nationals from African countries, by region of destination (1973–2013). Source: DEMIG VISA database

Figure 13 suggests that West Africa has become the most open to African migration on average, which seems to be linked to – at least nominally – free travel and migration between ECOWAS countries. Southern Africa has also become more open, which seems to be linked to the end of the *apartheid* regime which put a high priority in containing mobility from other African states. Over time, North Africa has shown a remarkable increase in visa restrictiveness for other African nationals from a comparatively low level of 69 % in 1973 to 89 % in 2013, which may partly reflect the political process of ‘externalisation’ of European border controls (cf. Betts & Milner, 2006; Paoletti, 2011). East Africa has staggering scores on the visa restrictiveness index for African nationals of between 80 and 90 %. Also Central Africa visa policies have also become more restrictive after an initial opening in the 1970s.

**Fig. 14:**
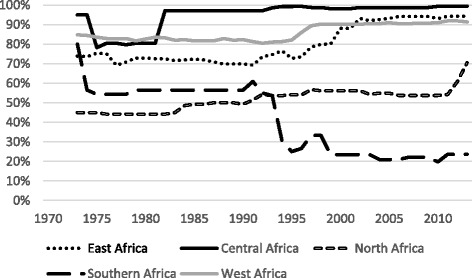
Visa restrictiveness of African countries for nationals from OECD countries, by region of destination (1973–2013). Source: DEMIG VISA database

Figure 14 specifies visa restrictiveness toward nationals from OECD countries^8^ for the different African regions. It shows that Southern Africa is the only African region that has become more open to OECD nationals over time, particularly after the end of the Cold War and the demise of the *apartheid* regime in the early 1990s, with an average of only 24 % of OECD countries needing a travel visa to enter Southern African countries in 2013, down from 80 % in 1973. This also seems to be in line with the idea that Southern Africa has the strongest global migration ties to its history of large-scale settlement of Europeans. In contrast, all other regions show an *increasing* visa restrictiveness for OECD nationals. While North Africa was relatively open in 1973 with a visa restrictiveness index score for OECD countries of 45 %, this had gone up to 70 % in 2013. Also East Africa is closing. Central Africa’s visa restrictiveness index for OECD citizens has been 99 % since the early 2000s. Interestingly, while West Africa has opened up to Africans citizens, it has become more closed to free travel of OECD nationals.

**Fig. 15:**
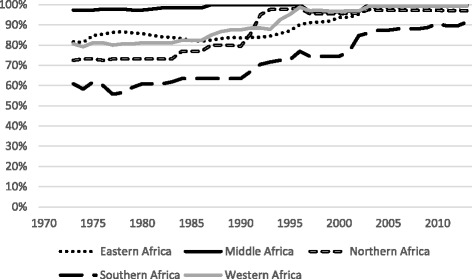
Visa restrictiveness of OECD countries for nationals from African countries, by region of origin of migrants (1973–2013). Source: DEMIG VISA database

It is not entirely clear which political processes explain the increasing visa restrictiveness of African countries towards OECD countries (with the exception of Southern Africa). One hypothesis is this partly mirrors the increasing visa restrictiveness of OECD countries for African citizens, suggesting that a certain degree of visa retaliation may have taken place. Figure 15 indeed confirms that there has been a massive trend of OECD countries closing their doors to free travel of African citizens, although there are clear regional differences. In 1973 OECD countries used to be relatively open towards nationals from Southern Africa and, to a lesser extent, from North Africa with restrictiveness indices of 61 and 73 % respectively. OECD visa restrictiveness for North Africans shot up since 1990 to reach 98 % in 1993, which seems to reflect the Schengen visa imposed for such countries in Europe. By 1995 all African regions had reached OECD visa restrictiveness scores of more than 90 %. For Southern Africans the increase was more gradual, reaching a value of 93 % in 2013.

Figures 13 and 14 show that, in overall, African countries have been more restrictive towards nationals from OECD and other non-African countries than towards African nationals. Yet the most striking finding is the extraordinary high visa constraints imposed on movement to and from African countries, whether within or outside of the continent, suggesting a high level of reciprocity in visa imposition. This particularly applies for Central, West and East Africa. It is not clear whether this reflects ‘retaliation’ or other processes. North and Southern Africa show different patterns. Although North African visa restrictiveness towards OECD citizens has increased, this does not match the almost near-universal closing of OECD countries towards North Africans. For Southern Africa, we even see a negative correlation as the region has become more open to travel from OECD citizens while reverse travel restrictions from Southern Africa to OECD countries have gone up.

**Fig. 16:**
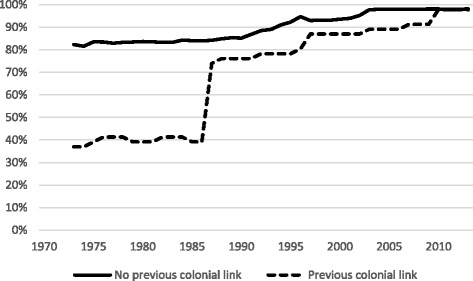
Visa restrictiveness of OECD countries for nationals from African countries, by type of link between countries (previous colonial link or not) (1973–2013). Source: DEMIG VISA database

The general increase in visa restrictiveness towards African citizens can be a partial driver towards an increasing spatial diversification of migration patterns away from colonial patterns. This reflects the broader argument that immigration restrictions change the character of migration rather than decreasing overall volumes of migration as such. Based on this, (De Haas, 2011) hypothesised four ‘substitution effects’ that can limited the effectiveness of immigration restrictions: (1) *spatial substitution* through the diversion of migration to other countries; (2) *categorical substitution* through a re-orientation towards other legal or illegal channels; (3) *inter-temporal substitution* affecting the timing of migration such as ‘now or never migration’ in the expectation of future tightening of policies; and (4) *reverse flow substitution* if immigration restrictions also reduce return migration, reduce circulation and thus push migrants into permanent settlement (see also Flahaux forthcoming). While all four effects may apply to African migration, the DEMIG VISA data seems to confirm the role of policies in encouraging spatial substitution in the form of diversion away from colonial pattern. Figure 16 shows that until the late 1980s former colonisers were much less restrictive towards migration of the nationals from previous colonial states. By 1988 this gap was largely closed, however, and in 2010 it was completely closed. This can partly explain the diversification of African emigration over the past few decades. So while increasing African emigration has been rooted in structural development trends, destinations seem to have been diversifying not because of a general fading of colonial links, but also partly as a result of closeness of former colonisers parallel to the more favourable immigration opportunities offered (particularly for the skilled) offered by new destinations such as the Canada, the US and Australia.

## Conclusion

Drawing on new data sources, this paper analyses the evolution of African migrations between 1960 and 2010. It particularly explores the role of development processes, state formation and policies in explaining trends and patterns of migration. Our analysis confirms that the majority of African migrants continue to move *within* the continent. Second, common perceptions of Africa as a ‘continent on the move’ are contradicted by the available data, which strongly suggests that the overall intensity of intra-African migration has decreased in recent decades, with the exception of West Africa. This decrease or – in the case of West Africa – stagnation of migration intensities may be related to state formation and the related imposition of barriers towards free movement in the wake of decolonisation as well as the concomitant rise of nationalism and related inter-state and intra-state tensions and violence. In this process, new independent states have tried to assert their newly acquired sovereignty by demarcating borders. Apart from the likely role of inter-state tensions in reducing unrestricted migration (such as in southern Africa in the apartheid era and considerably hostility between North African states), this also frequently coincided with considerable xenophobia and anti-immigrant sentiment in African societies. This is reflected in a high level of visa restrictiveness of African states for African nationals.

While the intensity of intra-African migration has decreased, there has been a recent acceleration and geographical diversification (beyond colonial patterns) of extra-continental migration from Africa to Europe, but increasingly also to North America, the Gulf and Asia. While most extra-continental migration once originated from North Africa, in recent decades there has been a rapid increased in migration from West Africa and, to a lesser extent, East Africa to wealthy countries outside Africa. This geographical diversification of African emigration seems partly connected to visa and other immigration restrictions put in place by traditional European destination countries (often former colonisers), and to a declining influence of the old colonisers and economic growth, labour demand and more liberal immigration regimes in the new destination countries. However, it is important to emphasise that the levels of extra-continental migration are still below those of migration within Africa and remain low for international standards. In the same vein as (Lessault & Beauchemin, 2009) argued, it would therefore be highly misleading to characterise migration from Africa as an exodus from Africa or an invasion of Europe and other destination countries.

Our analysis also contradicts conventional interpretations of African emigration as essentially driven by poverty. In general, African countries with comparatively higher levels of development (such as in the Maghreb or coastal West Africa) also tend to have the highest intensity of extra-continental migration, while the poorest countries (such as many landlocked Sub-Saharan countries) have lower levels of overall emigration and most emigration is dominated by short-distance migration to nearby countries. This seems to confirm the ‘migration transition theory’ (De Haas, 2010; Skeldon, 2012; Zelinsky, 1971), which argues that economic development and concomitant social transformation initially coincide with *increasing* levels and a larger geographical reach of emigration. Increasing income, education and access to information and networks generally increases people’s capabilities and aspirations to migrate. In sum, contradicting conventional interpretations of African migration being essentially driven by poverty, violence and underdevelopment, this paper shows that recent increases in African emigration should rather be explained from processes of development and social transformation which have increased young Africans’ capabilities and aspirations to migrate, a trend which is likely to continue in the future.

## Endnotes

^1^This term ‘environmental refugee’ (or ‘environmental migration’) is problematic in itself, as the impact of environmental factors on migration is highly dependent on many other political, economic and social factors. See also Gemenne (2011).

^2^'Africa' refers to the countries of the African continent and Madagascar. We took the decision to exclude the islands because of data inconsistencies.

^3^The regions named in this paper are those used in the classification of the United Nations (see map in Additional file 1).

^4^In 1960, 53 and 48 % of emigrants from Ethiopia and South Africa were estimated to live out of the continent, respectively.

^5^To Belgium, Denmark, Finland, France, Germany, Italy, Luxembourg, Netherlands, Norway, Spain, Sweden, Australia, New Zealand, USA and Canada.

^6^To Europe, North America and Oceania (Belgium, Denmark, Finland, France, Germany, Italy, Luxembourg, Netherlands, Norway, Spain, Sweden, Australia, New Zealand, USA and Canada)

^7^Because the values on this index were not weighed by the population size of origin countries, we can, strictly speaking, not state that a particular percentage of, for instance, ‘all non-African citizens’ need a visa to enter a particular country. The index therefore represent the number of countries for which a travel visa is imposed.

^8^We calculated this index for OECD countries separately, assuming that this group of relatively wealthy countries figure prominently among aspired migration destinations of inter-continental migrants, which is also confirmed by the analyses of stock in previous sections. The Gulf is the only major destination region excluded from this figures. The indices may be slightly misleading as African countries seem to be more open to free travel for European citizens than for OECD citizens in general.

## Additional files

Additional file 1: **Regions in Africa (UN classification).** (DOCX 24 kb)

Additional file 2: **Destinations of African migrants, by region of origin and continent of destination.** (DOCX 66 kb)

Additional file 3: **Evolution of continents of destination (for selected destination countries) by regions of origin of African migrants.** (DOCX 134 kb)
