# Supplementary material for: African migration: trends, patterns, drivers
Source: Commun Math Stat. 2016 Jan 22;4(1):1. doi: 10.1186/s40878-015-0015-6 (PMC4909155; doi:10.1186/s40878-015-0015-6)
Supplement: Supplementary file 1 — Regions in Africa (UN classification). (DOCX 24 kb) [file 40878_2015_15_MOESM1_ESM.docx]

## Additional file 1. Destinations of African migrants, by region of origin and continent of destination

| Destinations of East African migrants | Destinations of Central African migrants |
| --- | --- |
| 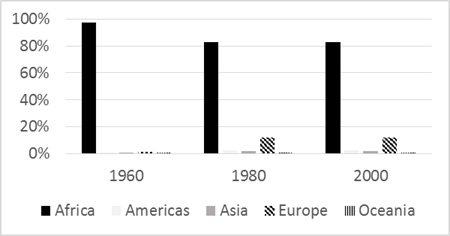 | 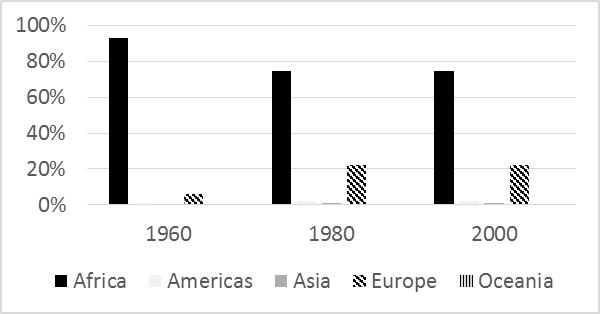 |
| Destinations of North African migrants | Destinations of Southern African migrants |
| 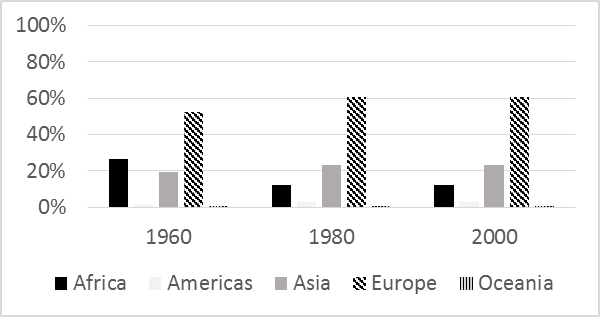 | 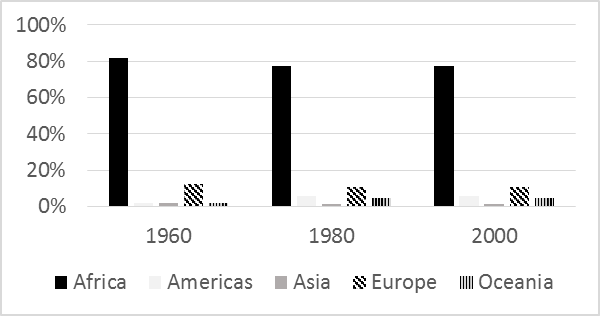 |
| Destinations of West African migrants |  |
| 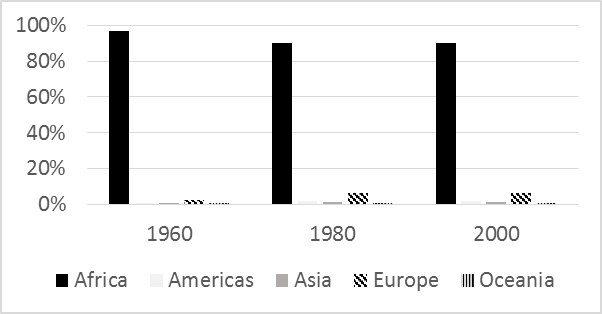 |  |

Source: Global Bilateral migration database
