# Supplementary material for: African migration: trends, patterns, drivers
Source: Commun Math Stat. 2016 Jan 22;4(1):1. doi: 10.1186/s40878-015-0015-6 (PMC4909155; doi:10.1186/s40878-015-0015-6)
Supplement: Supplementary file 2 — Destinations of African migrants, by region of origin and continent of destination. (DOCX 66 kb) [file 40878_2015_15_MOESM2_ESM.docx]

## Additional file 2. Evolution of continents of destination (for selected destination countries) by regions of origin of African migrants

| From East Africa (absolute numbers) | From East Africa (%) |
| --- | --- |
| 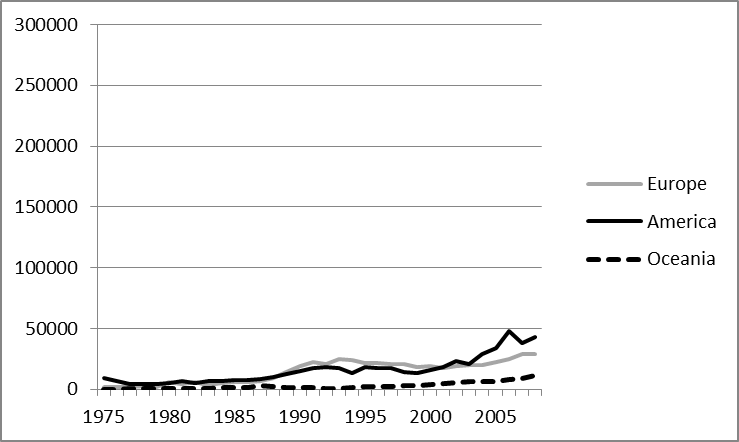 | 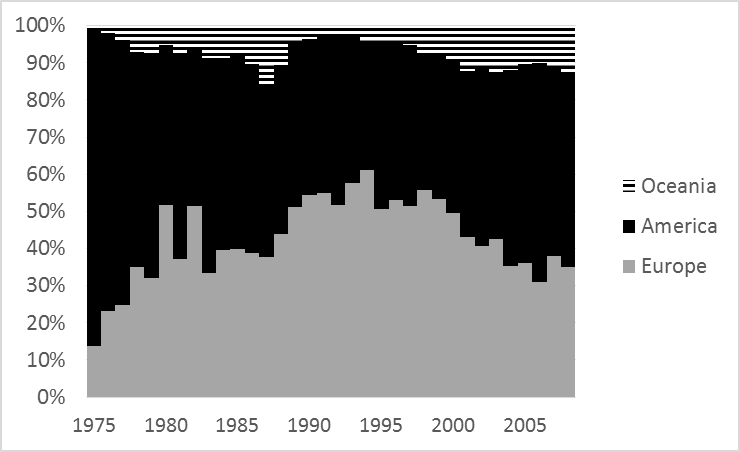 |
| From Central Africa (absolute numbers) | From Central Africa (%) |
| 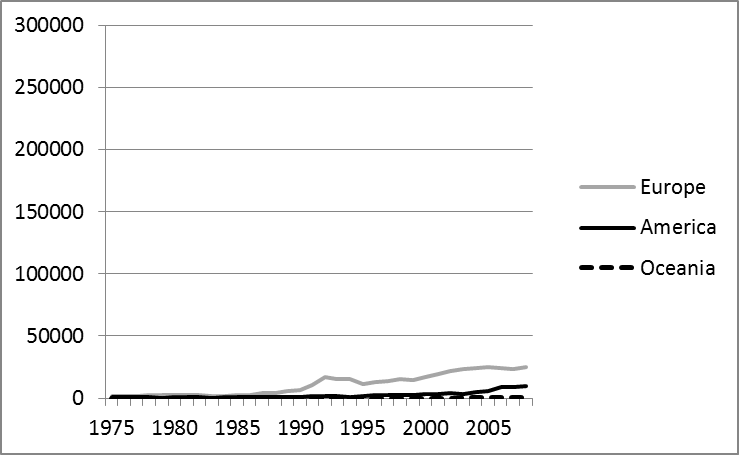 | 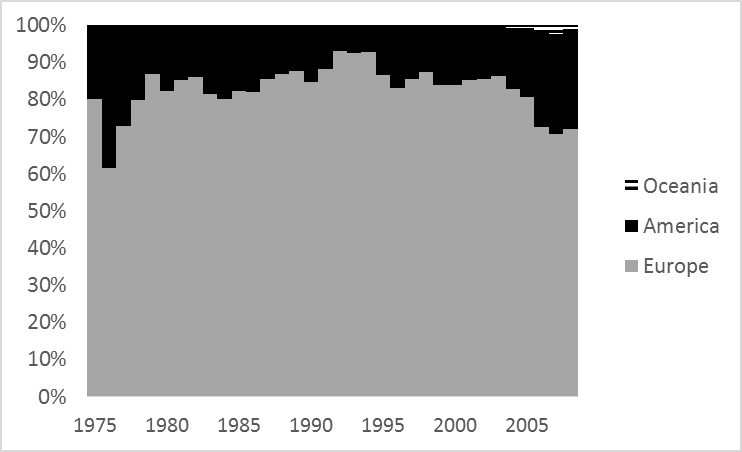 |
| From North Africa (absolute numbers) | From North Africa (%) |
| 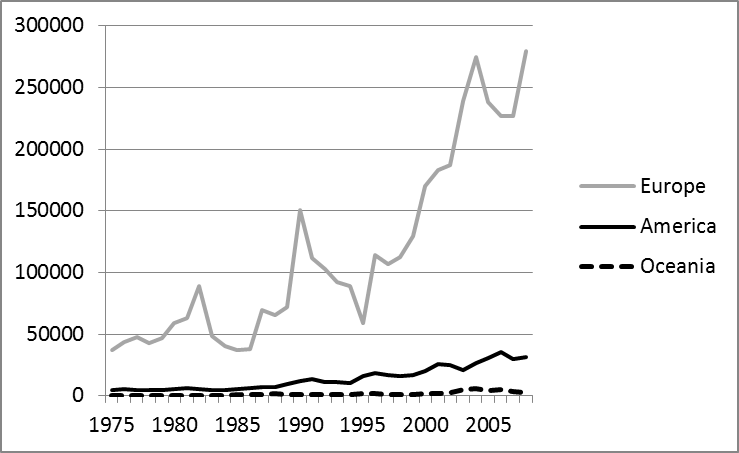 | 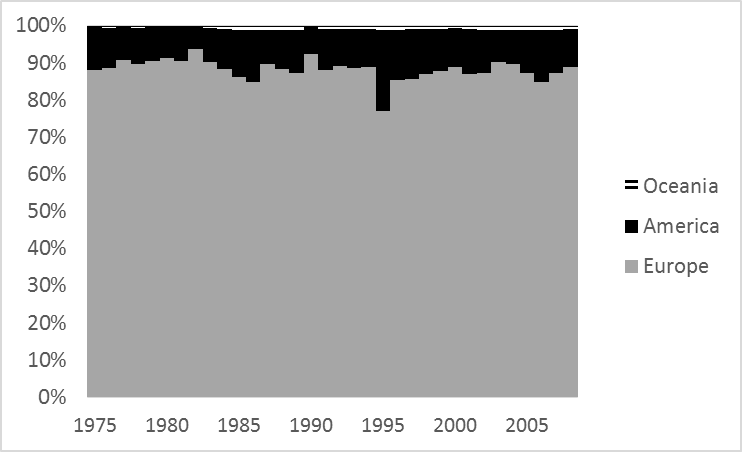 |
| From Southern Africa (absolute numbers) | From Southern Africa (%) |
| 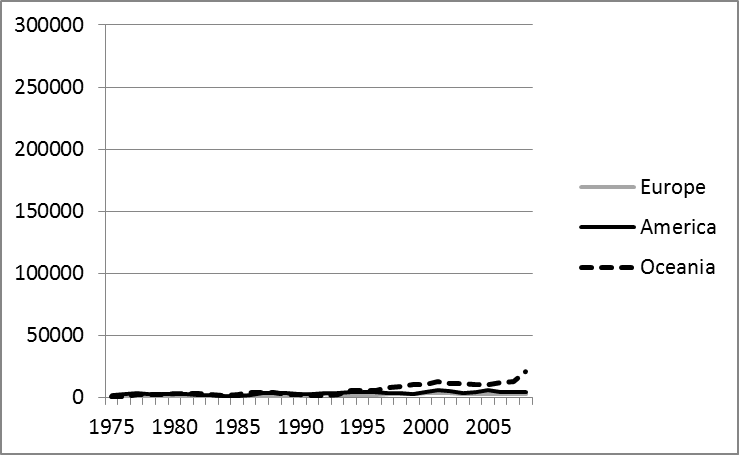 | 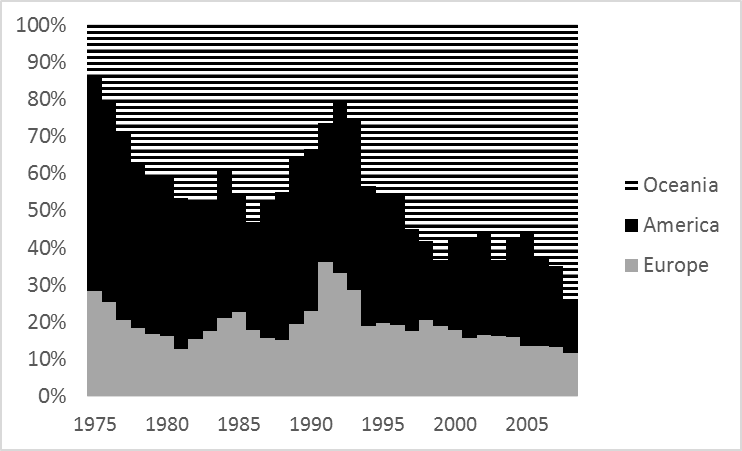 |
|  |  |
|  |  |
| From West Africa (absolute numbers)  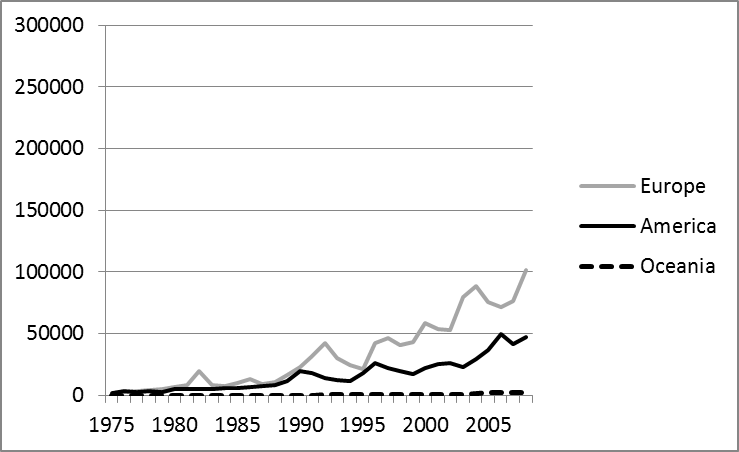 | From West Africa (%)  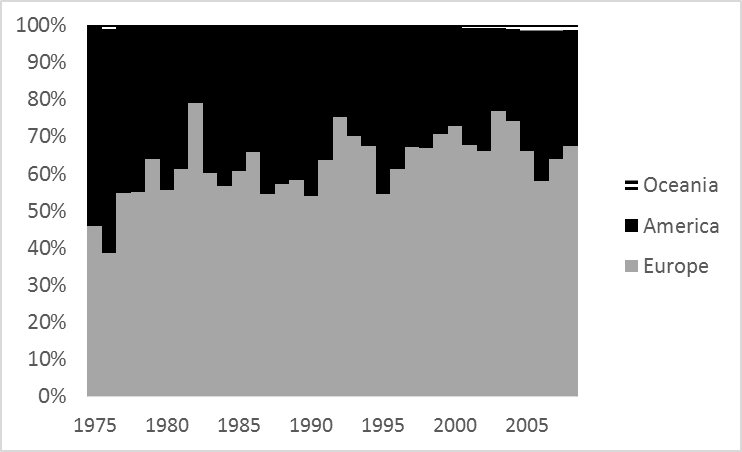 |

Source: DEMIG C2C database
