# Supplementary figures and images for: African migration: trends, patterns, drivers
Source: Commun Math Stat. 2016 Jan 22;4(1):1. doi: 10.1186/s40878-015-0015-6 (PMC4909155; doi:10.1186/s40878-015-0015-6)

## Additional file 3. Regions in Africa (UN classification)

**
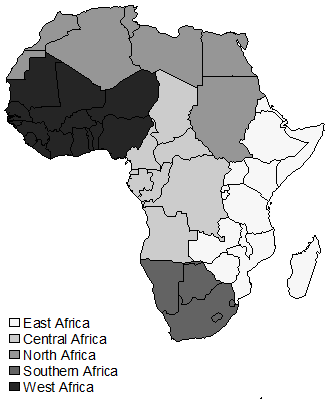
**

Supplement: Supplementary file 3 — Evolution of continents of destination (for selected destination countries) by regions of origin of African migrants. (DOCX 134 kb) [file 40878_2015_15_MOESM3_ESM.docx]
